# Innovative Assisted Living Tools, Remote Monitoring Technologies, Artificial Intelligence-Driven Solutions, and Robotic Systems for Aging Societies: Systematic Review

**DOI:** 10.2196/15429

**Published:** 2019-11-29

**Authors:** A Hasan Sapci, H Aylin Sapci

**Affiliations:** 1 Adelphi University Garden City, NY United States

**Keywords:** innovative assisted living tools for aging society, artificially intelligent home monitoring, older adults, robotic technologies, smart home

## Abstract

**Background:**

The increase in life expectancy and recent advancements in technology and medical science have changed the way we deliver health services to the aging societies. Evidence suggests that home telemonitoring can significantly decrease the number of readmissions, and continuous monitoring of older adults’ daily activities and health-related issues might prevent medical emergencies.

**Objective:**

The primary objective of this review was to identify advances in assistive technology devices for seniors and aging-in-place technology and to determine the level of evidence for research on remote patient monitoring, smart homes, telecare, and artificially intelligent monitoring systems.

**Methods:**

A literature review was conducted using Cumulative Index to Nursing and Allied Health Literature Plus, MEDLINE, EMBASE, Institute of Electrical and Electronics Engineers Xplore, ProQuest Central, Scopus, and Science Direct. Publications related to older people’s care, independent living, and novel assistive technologies were included in the study.

**Results:**

A total of 91 publications met the inclusion criteria. In total, four themes emerged from the data: technology acceptance and readiness, novel patient monitoring and smart home technologies, intelligent algorithm and software engineering, and robotics technologies. The results revealed that most studies had poor reference standards without an explicit critical appraisal.

**Conclusions:**

The use of ubiquitous in-home monitoring and smart technologies for aged people’s care will increase their independence and the health care services available to them as well as improve frail elderly people’s health care outcomes. This review identified four different themes that require different conceptual approaches to solution development. Although the engineering teams were focused on prototype and algorithm development, the medical science teams were concentrated on outcome research. We also identified the need to develop custom technology solutions for different aging societies. The convergence of medicine and informatics could lead to the development of new interdisciplinary research models and new assistive products for the care of older adults.

## Introduction

Life expectancy has increased worldwide, and countries have been experiencing the same challenges regardless of the geographical location. According to the US Census Bureau, the population aged ≥65 years is expected to double over the next three decades and reach 83.7 million [1]. One of the most significant challenges of the aging population is that the incidence of chronic conditions such as dementia, Alzheimer disease, congestive heart failure, and cancer and the need for medical attention have increased. However, rapid advances in technology have revolutionized medicine along with health care for the elderly.

Using a personal computer, remote patient monitoring device, smartphones, and mobile apps to improve the quality of the older persons’ lives was not an option in the past. Each new technology enabled researchers and clinicians to develop new disease management protocols, especially for frail elderly people with chronic diseases and dementia. Recent randomized controlled trials and systematic reviews have documented that remote monitoring reduces specific 30-day hospital readmission and mortality rates [2,3]. Aging in place is defined as “remaining living in the community, with some level of independence, rather than in residential care” [4]. Although technology usage is limited among seniors aged ≥75 years, several prototype and experimental systems were developed, and various studies were conducted to support the elderly by clinicians, computer scientists, data scientists, and engineers; however, few studies explored the current trends in senior care technology research [5]. Therefore, the aim of this study was to explore the current research trends and level of evidence for remote patient monitoring, smart home, and artificially intelligent monitoring systems.

## Methods

### Study Design

The literature search was conducted in February 2019. Remote monitoring and intelligent health care technologies research conducted in both health care and technology disciplines as well as the following literature repositories were chosen for the search: Cumulative Index to Nursing and Allied Health Literature Plus, MEDLINE, EMBASE, Institute of Electrical and Electronics Engineers Xplore, ProQuest Central, Scopus, and Science Direct.

A variety of synonymous terms were combined using Boolean logic, and a combination of three groups of keywords—(1) elderly, (2) home care, and (3) assistive technology—was selected as the keywords. To include all relevant publications, their thesaurus equivalent words and associated Medical Subject Headings terms—aging, aged, telemedicine, elderly people, nursing home, home health care, independent living, ambient assistive living, smart home technology, self-help devices, and artificial intelligence (AI) in older people’s care—were also included in the search (Table 1). We analyzed each article by the level of evidence and study type, objectives, and highlights. A combination of quantitative and qualitative approaches was used in the data analysis.

**Table 1:** Keywords and synonyms.

Keyword	Synonyms
Elderly	Aging (MeSH)^a^ or Aged (MeSH) or Elderly People
Home care	Nursing Home (MeSH) or Home Health Care or Independent Living (MeSH)
Assistive technology	Ambient Assistive Living or Smart Home Technology or Telemedicine (MeSH) or Assistive Technology or Self-Help Devices (MeSH) or Artificial Intelligence (MeSH) in Eldercare

^a^MeSH: Medical Subject Headings.

### Inclusion and Exclusion Criteria

Quantitative, qualitative, and mixed method peer-reviewed publications and published conference papers were included. Research articles and case reports related to assistive technology assessment for elderly care, set in homes, smart homes, experimental settings, nursing homes, and rehabilitation settings were selected.

The inclusion and exclusion criteria are listed in Textboxes 1 and 2, respectively.

**Inclusion criteria.**

- Published after January 2000 in English language
- Peer-reviewed journal articles and published conference papers
- Studies that focused on the latest technological, artificial intelligence, and complex software algorithms solutions for elderly care and novel assistive technologies and independent living
- Studies set in homes, smart homes, experimental laboratory settings, nursing homes, or rehabilitation settings
- Publications related to older people’s care, independent living, and novel assistive technologies

**Exclusion criteria.**

- Published before January 2000 in languages other than English
- Literature reviews and systematic reviews
- Book chapters, dissertations, theses, magazine articles, reports, wire feeds, position papers, editorials, white papers, and working papers

### Study Selection

**Figure 1:**
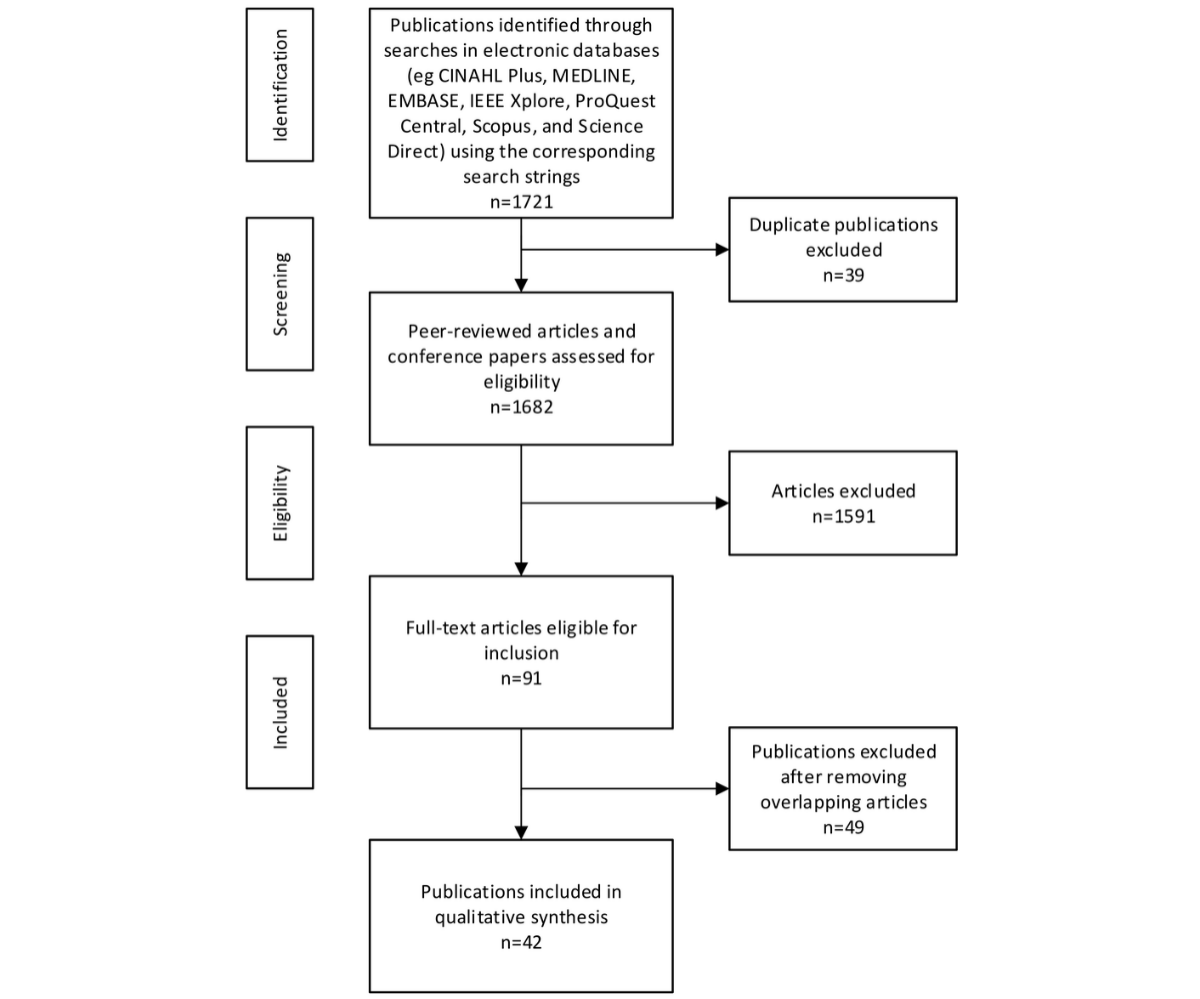
Search methodology. CINAHL: Cumulative Index to Nursing and Allied Health Literature; IEEE: Institute of Electrical and Electronics Engineers.

A total of 1721 publications were found at the end of the initial search of the selected databases. Search strings and return values for each database are listed in Textbox 3 and Table 2, respectively. The list was filtered by removing duplicates, the remaining abstracts were assessed, and the publications that did not meet the inclusion and exclusion criteria were excluded. At the end of this process, 91 eligible publications for inclusion were identified. Figure 1 displays the search diagram and the number of articles assessed at each stage of the review.

**Search string.**

(“Aging” OR “Aged” OR “Elderly People”) AND (“Nursing Home” OR “Independent Living”) AND (“Self-Help Devices” OR “Telemedicine” OR “Ambient Assistive Living” or “Service Robot”)

**Table 2:** Search queries and return values.

Database name	Return value (n)
Cumulative Index to Nursing and Allied Health Literature Plus	93
MEDLINE	159
EMBASE	279
Institute of Electrical and Electronics Engineers Xplore	31
ProQuest Central	747
Scopus	352
Science Direct	60

## Results

Analysis of the 91 full articles revealed innovative technologies that were developed to monitor older people’s activities using various sensors, telemedicine, assistive robots, and remote monitoring devices. Multimedia Appendix 1 lists the characteristics and highlights of the included studies [6-86].

Our review determined that the studies either focused on technology acceptance or examined the development of new patient monitoring and smart home technologies, real-time transmission of raw data, and AI algorithms. In the publications reviewed, most articles were qualitative, and only five studies were randomized controlled trials. First, we categorized the articles by types of study design. Most studies were quasi-experiment (n=43) and case reports (n=39). The others were case-control studies (n=3), cohort studies (n=1), and randomized controlled trials (n=5). The number and percentage of these articles are listed in Table 3.

Second, we evaluated the levels of evidence of each article. The Oxford Centre for Evidence-Based Medicine levels rates evidence based on the study design, rigor, and validity and judges the strength of evidence in a technically accurate and easily understandable manner. Therefore, this well-established and accepted standard was selected to determine the level of evidence [87]. The majority of the articles were listed as Level IV evidence that represents poor or nonindependent reference standards (49/91, 49%). Only 6% (6/91) of articles were listed as Level I evidence, which represents the studies with good reference standards. The classification of publications by levels of evidence is shown in Table 4.

**Figure 2:**
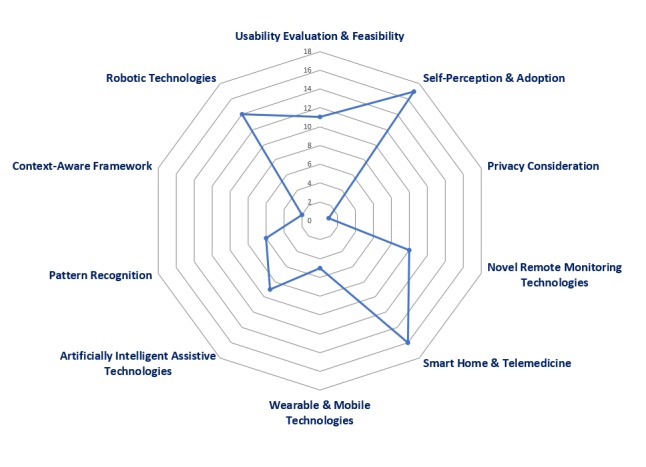
Study by research focus.

Third, to determine the current status and future challenges of disruptive technologies to support independent living, the selected articles were analyzed with regard to study objectives. Figure 2 summarizes the focus of the articles evaluated. Of 91 articles, the majority were focused on older adults’ acceptance and adoption of monitoring technology (n=17), smart home and telemedicine apps (n=16), robotic technologies (n=14), and usability evaluation (n=11). Many researchers evaluated novel remote monitoring technologies (n=10) and artificially intelligent assistive technologies (n=9). The remainder of the publications were about pattern recognition (n=6), wearable and mobile technologies (n=5), context-aware framework (n=2), and privacy considerations (n=1).

Fourth, as there is no widely accepted classification system to evaluate elderly care research that focuses on technology solutions, we conducted a thematic analysis, removed overlapping articles, identified 42 publications, and analyzed each device and app. Braun and Clarke [88] thematic analysis method was used to determine the patterns.

Finally, the studies that represent evolving topics were identified, and four themes emerged: technology acceptance and readiness, novel patient monitoring and smart home technologies, intelligent algorithm and software engineering, and robotics technologies (Multimedia Appendix 2) [6-29,56,57,65,73,103].

**Table 3:** Study types (N=91).

Category	Articles, n (%)
Case report	39 (42)
Case-control study	3 (3)
Cohort study	1 (1)
Quasi-experiment	43 (47)
Randomized controlled trial	5 (5)

**Table 4:** Levels of evidence (N=91).

Category	Articles, n (%)
I	5 (5)
II	6 (6)
IV	45 (49)
V	35 (38)

### Technology Acceptance and Readiness

### Overview

This theme encompasses studies related to technology adoption. Researchers investigated older adults’ willingness to accept contactless monitoring technologies, specific electronic health system, and smart home–based technologies such as bed, motion, kitchen safety, and fall detection sensors [6-8].

In addition to technology adoption, some studies explored perceived usefulness of telehealth kiosk—telehealth care systems that measure bed and chair occupation and detect falls, privacy concerns of in-home monitoring systems, older people’s attitudes toward assistive telemonitoring systems, acceptability of home monitoring technologies, and video-based monitoring technologies that capture data about daily activities [9-89]. Caregivers’ acceptance of home telecare technologies was also investigated along with wearable and ambient technologies [14,15].

### Novel Patient Monitoring and Smart Home Technologies

This theme comprises sophisticated systems that consist of home service robot; home and body sensor network; mobile device; cloud servers and remote caregivers; supervised machine learning approach and context-based reasoning to perform a clinical assessment of dementia; proof-of-concept platforms that consists of a Zigbee network, sensors, a home client, and remote server; and novel protocols over SMS to monitor elderly and alert caregivers when a fall occurs [90-92].

**Figure 3:**
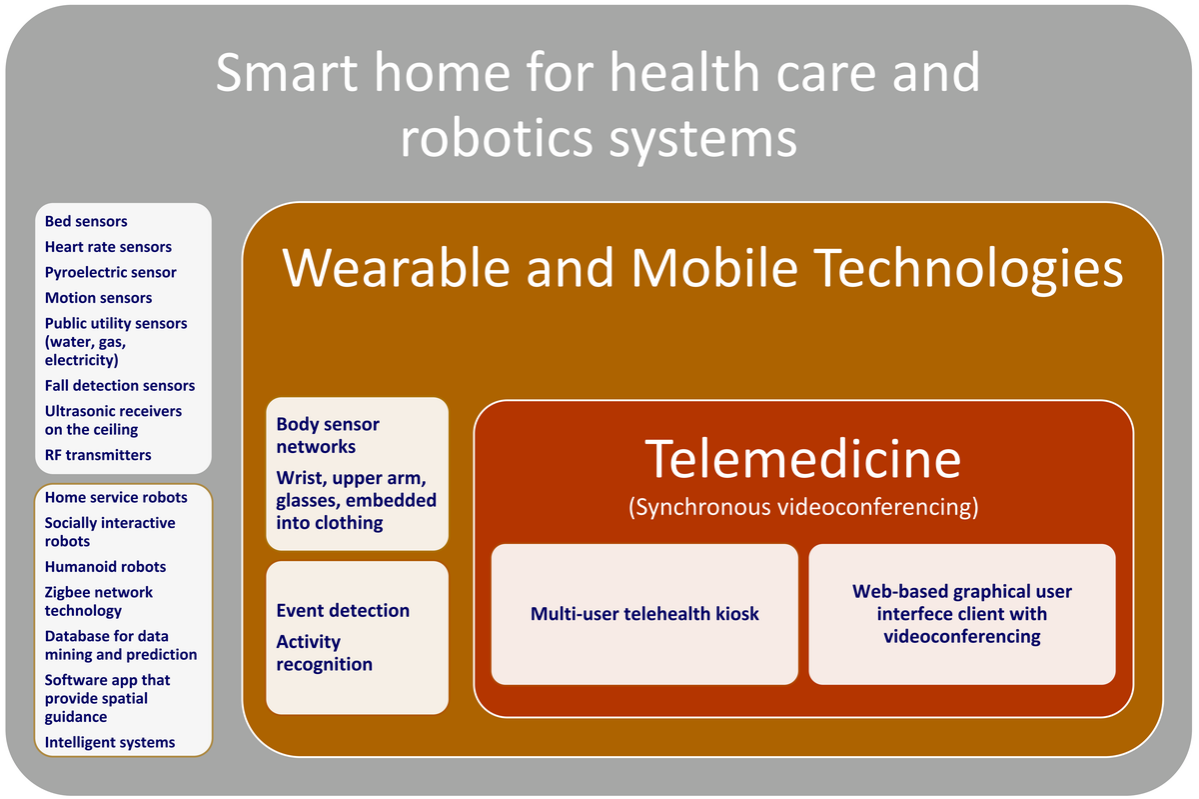
Technologies used for aging societies. RF: radio frequency.

Common denominators for smart home for health care, robotics, wearable and mobile systems, and telemedicine apps were determined by analyzing each device and app. Our study revealed that researchers who focused on smart homes preferred novel sensor systems and ultrasonic receivers and transmitters for their study, and those who focused on remote monitoring preferred custom wearable devices and telemedicine equipment (Figure 3).

In Europe, an international experimental Ambient Assisted Living project called Enhanced Complete Ambient Assisted Living Experiment successfully tested fall detection, activity classification, and energy expenditure algorithms to monitor patients’ activity and reduce morbidity and mortality [17]. Another novel technology explored by researchers was virtual reality (VR), and new technologies about the display quality, presence, user input, fidelity, and usability of virtual experiences demonstrated effectiveness to assess functional behavior and emphasized the potential of VR technology to empower dementia patients [18].

Several experimental prototype devices were also developed to monitor elderly patients’ progress and treatment. With regard to innovative low-cost Bluetooth-enabled technologies, some researchers developed a telediagnosis system for early detection of Alzheimer disease and captured the movement patterns [93]. A Web-based home monitoring system using wearable sensors was developed for patients with Parkinson disease [94].

A randomized controlled trial was designed to evaluate the Integrated Telehealth Education and Activation of Mood project’s clinical outcomes and demonstrated that the integration of telemonitoring intervention improved geriatric home care patients’ problem-solving skills and self-efficacy in managing their chronic illness [19]. In a different study, a sensor network system that comprised ultrasonic receivers, signal generators, radio frequency transmitters, ultrasonic 3D tags, and a computer successfully detected the accident-prone events in advance [95].

**Figure 4:**
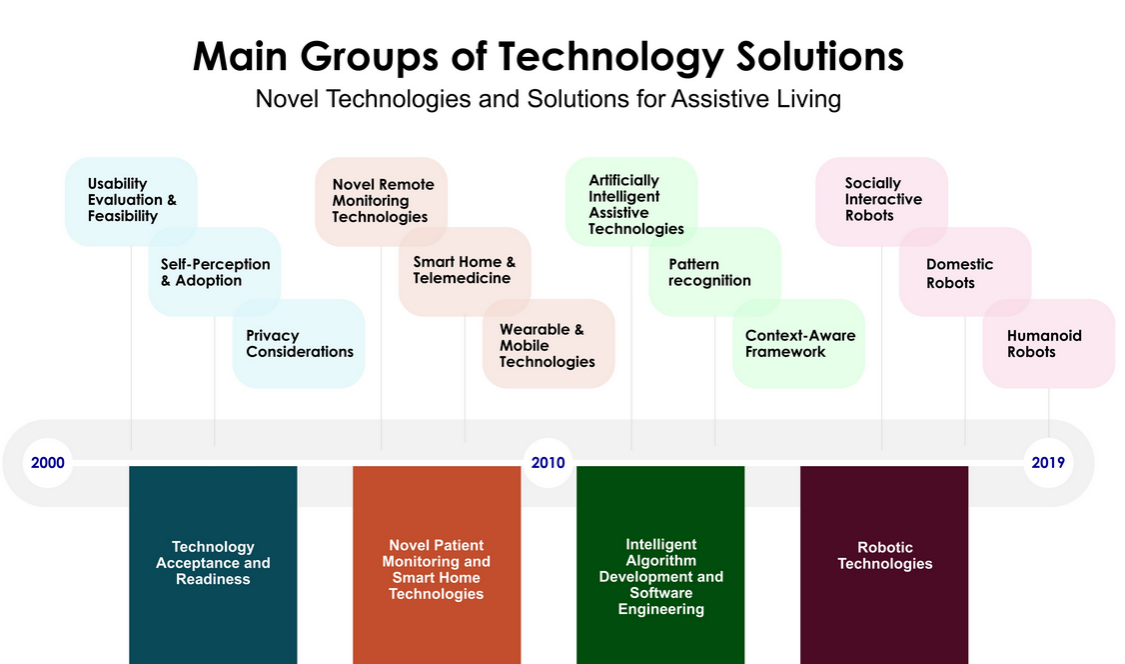
Technology solutions for elderly care.

The increasing availability of the broadband internet, cellular communication technologies, internet of things apps that connect multiple devices and the decreasing cost of sensors have transformed various industries and markets. Our study demonstrates the potential of novel platforms that can improve assisted living and elderly care (Figure 4).

### Intelligent Algorithm Development and Software Engineering

This theme comprises data mining algorithms that collect data about the environment and intelligently predict possible problems to make health care decisions, context-aware middleware to sense and respond to the user’s environment, pyroelectric sensors, and infrared optoelectronic components designed to detect electromagnetic radiation and analyze the reasoning process in order to detect elderly people’s activity [21-96].

Some researchers developed predictive models and reported the optimal classifier of assistive technology adoption for people with dementia [22]. Another proof-of-concept navigation system based on augmented reality successfully generated a route to a specific destination based on the user context including well-known places, social relationships, and point of interest (Figure 4) [23]. In information science, the term *ontology* encompasses entities, relations, functions, axioms, and instances. Ontology-based models combine data from multiple sources [97]. Researchers designed and successfully tested an ontology-based prototype knowledge system that can collect data from an RGB camera, 3D depth camera, and microphones [98].

**Figure 5:**
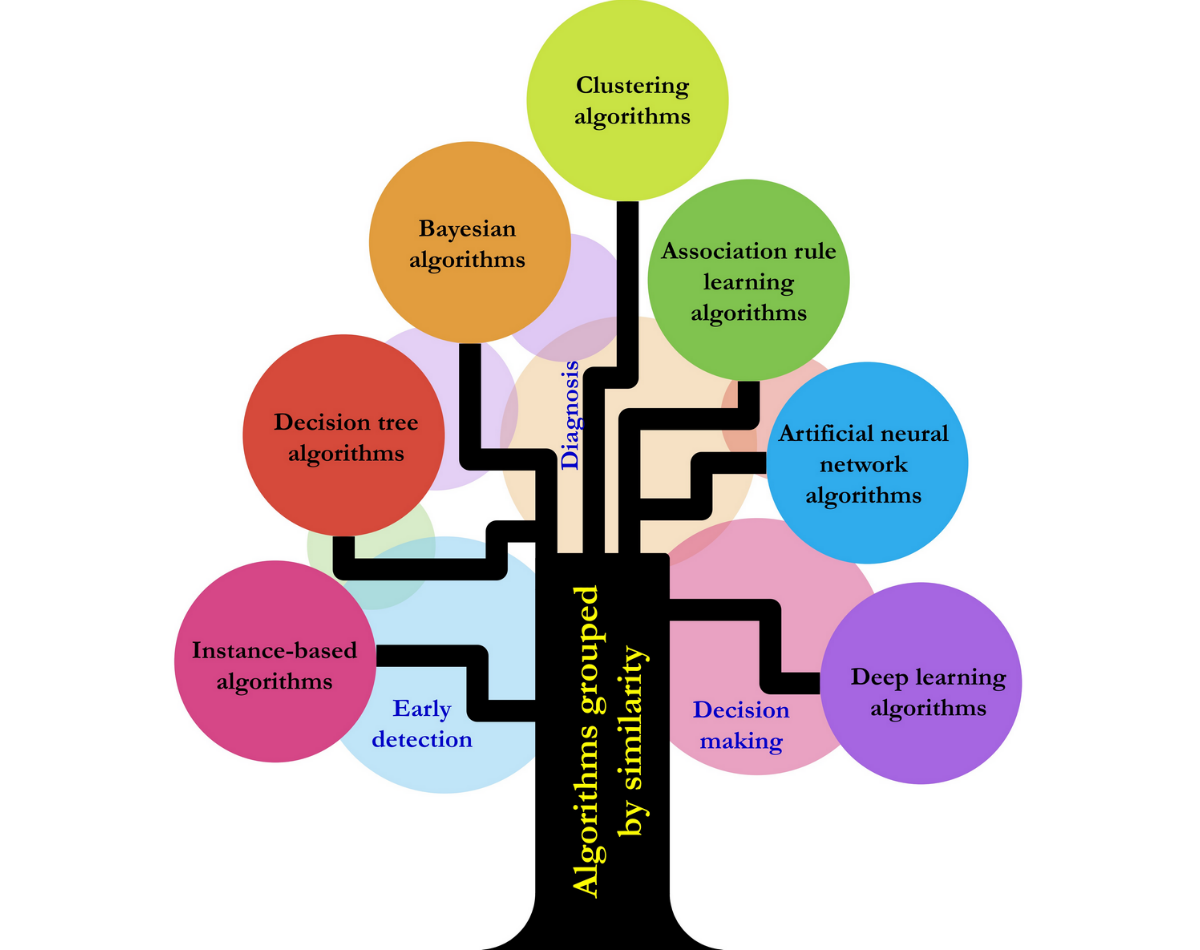
Artificial intelligence algorithms for independent living.

Figure 5 summarizes the AI algorithms used for independent living apps. Most AI studies focused on instance-based algorithms, decision tree, Bayesian algorithm, clustering, association rule learning, artificial neural network, and deep learning algorithms.

### Robotics Technologies

This theme encompasses various robotic technologies that affect elderly care. Although some studies investigated a range of advanced AI technologies [24,99], others examined simple versions to evaluate robot-assisted activities. The increasing number of individuals that require rehabilitation and assistance has driven innovators to develop new robotics systems that can be integrated with elderly care solutions. Telepresence, companion, home automation and service, rehabilitation and health monitoring, and reminder robots can assist individual living.

The researchers who assessed AI technology for domestic assistive services developed eight different scenarios to understand the usefulness of the domestic robot in everyday situations and partially validated their results with experiments involving 100 participants [100]. Other researchers who evaluated robotic technologies used commercially available robots with tactile, light, and posture sensors and focused on studies about socially interactive robots. Similarly, a team of researchers in Japan developed a partner robot to relieve the solidarity feeling of elderly through conversation, quizzes, tongue twisters, and arithmetic calculations, and the experimental results were found to be promising [25].

Telepresence robots are remote-controlled devices that a user can drive from a different location and communicate with a remote site using the integrated videoconferencing systems.

A team of researchers used a sensor network infrastructure that comprised pressure monitor, glucose, weight, and oxygen sensors integrated with a telepresence robot. The project received support from the European Commission and deployed in real homes across Europe [26]. Another multidisciplinary effort to develop a mobile robotic assistant was the Pearl project. Researchers from the University of Michigan, University of Pittsburgh, and Carnegie Mellon University developed an autonomous robot to provide cognitive orthotic functions and tested it in a residential retirement community successfully [27].

Interestingly, some studies did not find any difference between a therapeutic robocat and plush cats, and others emphasized technical challenges of intelligent modular service mobile robots that comprised tactile, infrared, and ultrasound sensors; Kinect and voice generation; and recognition systems [24,101] Robots may also not be the best solution for certain care-related activities. A remote-controlled Spykee robot was used to make home hazard assessments for fall preventions, and it did not find an agreement between the robot and in-person video assessment [28]. A recent study conducted in Europe evaluated a software framework’s efficiency using a humanoid robot and validated each solution’s efficiency using simulation and real case experiments [99].

This study revealed the multidisciplinary aspect of robotic technology and the development of autonomous mobile robots that can interact with elderly people and provide therapeutic benefits. The implementation of autonomous robots in elderly care requires collaboration among academic institutions, clinicians, and industry players and a focus on continuously improving the health care experience.

## Discussion

### Principal Findings

The purpose of this literature review was to determine the best available evidence about the development and implementation of technological solutions for elderly care, and in this paper, we report that the evolving technology trends can transform the aging population and ways that AI and pattern recognition might impact older individuals’ care.

Specifically, we examined publications about intelligent remote monitoring, smart home health care, and robotic technologies to respond to the following research questions: (1) *what are the current trends in aging-in-place technology research?* and (2) *what is the level of evidence for remote patient monitoring, smart home, and artificially intelligent monitoring systems?*

To answer the first question, we determined the wide range of studies that focused on technology acceptance, novel patient monitoring and smart home technologies, intelligent algorithm development and software engineering, and robotics technologies. To answer the second question, the breakdown of the articles identified that most studies (85%) had poor reference standards without an explicit critical appraisal (Table 3), and the majority of the publications were qualitative.

This literature review demonstrated that most studies between 2000 and 2010 were designed to examine older adults’ perceptions of technology. Intelligent assistive technologies have changed with an unpredictable pace, and consequently, there has been an increasing interest in exploring patient monitoring and home care technologies. The studies about technology acceptance led to more sophisticated studies that used wireless monitoring devices, sensors, intelligent algorithms, and experimental or quasi-experimental research methods. Thus, these studies can be considered as the first era of technology research for the aging society (Figure 4).

After 2010, we noticed an increase in the number of studies that explored prototype system development, implementation of new smart home technologies using sensors, development of assistive robots, and design of new AI and machine learning systems to support elderly care. The advancements in technology gave the researchers the ability to develop sophisticated AI algorithms, integrate advanced context acquisition methods, and analyze and automate high-level and complex tasks. This period can be considered as the second era of technology research for aging society, as many studies documented the potential use of robotic technologies, reported encouraging adoption rates, and recommended further experimental studies.

Our analysis demonstrated that many of the studies used unique technological solutions for different elderly groups. For example, studies that support independent lifestyle were designed for older people living alone in their home, whereas studies about new technologies for dementia and patients with Alzheimer disease were designed for older people living in nursing homes.

Data generated from medical devices have been growing so fast that using manual techniques to analyze data is not an option anymore to monitor home care patients. A recently published study assessed the use of patient-generated data in clinical practice and emphasized its impact on health outcomes [102]. Over the last decade, there has been a significant increase in the number of studies that focused on AI and machine learning. Some studies investigated user perceptions, barriers, and novel system development using sensors and smart home devices, whereas others focused on the development of context-aware and adaptive technology development. This technology can be integrated into different environments; can collect specific information such as temperatures, geographic locations, and user preference; and can deliver the relevant data depending on a set of variables unique to the user. Our review also revealed that the focus of AI apps for elderly care and sophisticated algorithms could improve the accuracy and the progress of analytical techniques. Therefore, it is likely that when combined with AI apps, remote monitoring systems will work faster and make more accurate predictions.

Moreover, this study identified several studies about novel innovative systems to monitor older people’s health. Many of these studies were proof-of-concept systems to demonstrate the feasibility of the proposed equipment or app. It is quite challenging to determine the benefits and long-term impact of each technology or prototype systems because some technologies might become widely adopted in time, whereas others cannot find enough support for implementation. Furthermore, designing studies to validate health care institutions’, nursing homes’, and individual patients’ technology adoption rates for elderly care might be challenging. Thus, we recommend nationwide studies to monitor technology adoption trends. Although this is an ambitious objective for individual researchers, governments and academic research institutions can collaborate and conduct these studies.

### Limitations

This study has some limitations. First, most study findings were not comparable because of the various research settings and types of technology used. Second, the majority of the studies were uncontrolled and had small user groups, and their level of evidence was between IV (45/91, 50%) and V (35/91, 39%). Owing to small sample sizes and methodological weaknesses in the studies, it was difficult to generalize their outcomes.

### Conclusions

**Figure 6:**
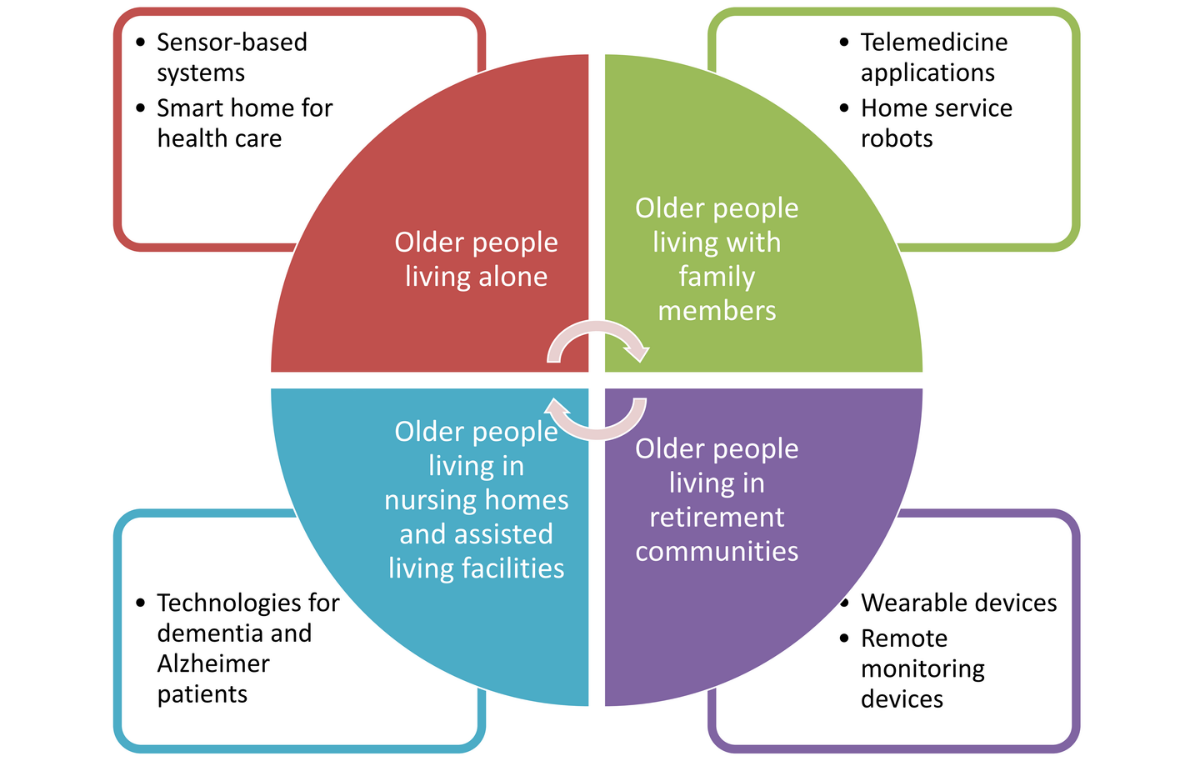
Technology solutions for different aging societies.

Medical and engineering sciences have different principles and use different approaches for assisted living, home care, and telecare innovations. It is probable that older people’s care will rely more on technology-driven patient solutions and AI algorithms to determine early warning predictions and initiate the interventions at earlier stages. Hence, we also propose the development of custom technology solutions for different aging societies: (1) novel smart home apps and sensor-based systems for older people living alone, (2) home service robots and telemedicine apps for older people living with family members, (3) wearable and remote monitoring devices for older people living in retirement communities, and (4) technologies to assist older people with dementia living in nursing homes and assisted living facilities (Figure 6). Machine learning and AI might be embedded into any hardware device, and further study is needed to identify aging society’s custom technological needs and determine AI research priorities. Taking into consideration different aging societies’ custom needs will improve older people’s independent living skills and elderly patients’ health care outcomes.

## Appendix

Multimedia Appendix 1 Characteristics of included studies.

Multimedia Appendix 2 Emerging technology solutions.

## Abbreviations

AI: artificial intelligence
VR: virtual reality
