# Supplementary material for: Innovative Assisted Living Tools, Remote Monitoring Technologies, Artificial Intelligence-Driven Solutions, and Robotic Systems for Aging Societies: Systematic Review
Source: JMIR Aging. 2019 Nov 29;2(2):e15429. doi: 10.2196/15429 (PMC6911231; doi:10.2196/15429)
Supplement: Multimedia Appendix 1 [file aging_v2i2e15429_app1.docx]

| **Author [reference]** | **Year** | **Level of evidence^a^** | **Study type** | **Study objectives** | **Highlights** |
| --- | --- | --- | --- | --- | --- |
| Chivarov et al [36] | 2014 | V | Case report | Development of intelligent modular service robot for disabled elderly people care | Intelligent modular services robot will serve food and drink, remind medications, turn on electric devices, and alert physician depending on patients’ health status. |
| Zhang et al [32] | 2014 | V | Case report | Evaluation of predictive adoption models for home-based care for people with dementia | The predictive model derived from a k-nearest-neighbors algorithm using 7 features is the optimal classifier of assistive technology adoption and can maximize the improve elderly people's assistive technology adoption. |
| Wild et al [45] | 2008 | IV | Case-control study | Identify in-home monitoring needs and expectations of older adults | This study evaluated older adults’ reactions for continuous in-home monitoring and determined the dominant themes as independence, cognitive decline, privacy, and information sharing. The researchers determined that the acceptance was closely tied to perceived utility of the system. |
| Osamu et al [46] | 2014 | V | Case report | Development of a prototype patient monitor system using AI^b^ for dementia and cognitive impairment caregiver support | The proposed intelligent caregiver system can identify and monitor cognitively impaired patients, assess risks, and alert the caregiver via alarm, text, or voice. |
| Wang et al [47] | 2014 | V | Case report | Implementation of intelligent caring agents for wellness monitoring for aging-in-place | Intelligent agents can communicate with each other, exchange information, detect inconsistencies in daily activities, provide supportive dialogue, and accompany elderly people at home. |
| Di Lecce et al [48] | 2015 | V | Case report | Development of an ICT^c^ tool to monitor social isolation level for elderly people with sensory and neuromotor disabilities | The proposed system measures social isolation using an AI approach on learning capacity and interest related to new technologies. |
| Ge et al [31] | 2015 | V | Case report | Development of an intelligent reasoning machine and remote monitoring app to detect elderly people’s regular activities | The proposed system was tested in a laboratory environment. The reasoning machine provided accurate alarms except 1 event during the 20-day trial period. |
| Kurnianingsih et al [30] | 2015 | V | Case report | Development of an ontology-based context-aware framework to monitor elderly people’s biological data, medical problems, decreased body metabolism, and daily activity for ubiquitous home care | Ontology-based system contains a personalization option to learn the subsystems. |
| Wilson et al [49] | 2016 | V | Case report | Development of analogical reasoning and decision theory of an AI system to assist elderly people and provide professional care | The proposed socially assistive robot will interact with elderly people and help with medication management tasks. |
| Amiribesheli et al [50] | 2016 | V | Case report | Accommodation of specific needs of people with dementia and the development of a dementia-friendly smart home prototype | This study describes the development of a prototype system that comprises the following 5 phases: phase 1—studying the symptoms and forming the preliminary scenarios; phase 2—scenario refining and initial evaluation; phase 3—requirements elicitation; phase 4—prototype implementation; and phase 5—prototype evaluation. |
| Kurnianingsih et al [51] | 2016 | V | Case report | Comparison of 5 supervised learning algorithms to monitor the position of elderly people and predict their next position | The authors present a predictive positioning system. The researchers compared 5 algorithms and determined that the deep learning provided best results to monitor the elderly people’s position inside home. |
| Yassine et al [52] | 2017 | V | Case report | Development of a model to recognize human activity patterns for low-resolution smart meter data and the detection and prediction of behavioral abnormality using smart home technologies | The proposed model used a dataset that included 400 million smart meters raw records to extract frequent patterns and track the well-being of individuals living alone. The study demonstrated high accuracy of the proposed model to correctly detect multiple appliance usages and make short- and long-term predictions. |
| Yang et al [53] | 2017 | V | Case report | The implementation of humanoid robots to make appropriate decisions about elderly people care through reinforcement learning with human feedback | Pepper, the humanoid robot, was tested to recognize face, greet the incoming person, answer some basic questions, track the repetitive utterance, and calm down the patient when needed; the experiment result showed a promising performance. |
| Elakkiya et al [20] | 2017 | V | Case report | Assessment of dementia patients’ context-based clinical score assessments to monitor routine activities and the development of a hybrid assessment system for dementia care through smart home | The experimental assistive health care system for dementia has shown 80% accuracy. |
| Pirzada et al [54] | 2018 | V | Case report | The design and implementation of a sensor-based smart home environment that can identify and predict problems by monitoring daily living activities | The proof-of-concept study detected anomalies using offline data. |
| Jeon et al [35] | 2018 | V | Case report | Design and test of an ontology-based knowledge system to provide care for people with dementia | The ontology-based knowledge system adaptively adjusted depending on the assessment context of the person. The proposed system can be used with knowledge sharing systems and robot platforms. |
| Suryadevara et al [55] | 2012 | V | Case-control study | Detection and analysis of elderly people activity behavior and the development of an intelligent, low-cost, real-time home monitoring unit for elderly people | The developed system continuously monitors elderly people’s activity based on the usage of the household appliance and generate daily activity pattern. The generated activity pattern can be used to predict abnormal behavior. |
| Wilson et al [56] | 2019 | V | Case report | Integration of a robot into smart environment to provide interactive support to individuals with functional limitations and evaluation of the robot activity support system to detect and map objects | This project describes components and demonstrates a robot activity support system that links smart home devices to physical robots to support daily activities. |
| Ramoly et al [37] | 2018 | V | Case report | The development and validation of an uncertainty management framework for humanoid robots | The proposed software framework acquired context data from the robot and smart environment sensors; analyzed and determined outdated, inexact, partially wrong, and contradictory information; and achieved 90% correct recognition. Future studies will improve the decision-making process. |
| Ferreira et al [22] | 2013 | V | Case report | The development of an Android mobile app for elderly care. The app can send fall alerts, medicine and activity reports, and location information. | The proposed system was designed to improve elder-caregiver communication using a smartphone app. During the development phase, the authors considered usability issues associated with elders, such as the difficulty to distinguish between long and short screen presses and the use of large and colorful buttons. |
| Cook [29] | 2006 | V | Case report | The development of agent-based smart home technologies | The proposed system identified lifestyle trends, detected anomalies, and automated assistance. |
| Libin and Cohen-Mansfield [42] | 2004 | IV | Case-control study | The use of robotherapy as an artificial companion for elderly people | The study evaluated cognitively impaired nursing home residents’ interactive sessions with robotic cat NeCoRo. Agitated behaviors were assessed on the Agitated Behaviors Mapping Instrument. The study demonstrated that the robotic cat produced significant increase of pleasure. The authors also compared plush cat and concluded that although there was plash, cats did not display any interactive behavior; there was no difference in using either a plush or robotic cat. |
| Tamura et al [57] | 2004 | IV | Case-control study | The use of toy robot for occupational therapy for elderly people with dementia | The entertainment robot triggered memories of past feelings of comfort and was evaluated as a therapeutic tool for elderly people with dementia living in geriatric homes. |
| Suryadevara et al [58] | 2013 | V | Case report | The development of a novel behavior detection process to forecast elderly people’s behavior and wellness by monitoring the daily usages of appliances in a smart home | The proposed prototype can collect data, analyze it in real time, evaluate the basic wellness parameters using sensing and intelligent behavior detection subsystems, and determine the trend about the elderly people’s daily activities. |
| Vincent et al [59] | 2006 | II | Quasi-experiment | Quasi-experimental study; the evaluation of a telesurveillance system’s effectiveness for the elderly | Telesurveillance is the continuous observation of patients without the transmission of any physiological data. This study evaluated telesurveillance system’s effect over 9 months using personal questionnaires and did not find any change in elders’ quality of life and daily activity abilities. The satisfaction was very high. |
| Pecina et al [60] | 2013 | I | Randomized controlled trial | Randomized controlled trial of daily home monitoring; patients aged ≥60 years were randomly selected to follow up via usual care and daily home telemonitoring for 1 year | At-risk older adults with higher Elderly Risk Assessment score were followed with the Intel Health Guide. Their blood pressure, weight, pulse, temperature, pulse oximetry, and peak flows were monitored daily at home. Home telemonitoring did not improve self-perception of mental well-being. |
| Kearns et al [61] | 2012 | II | Cohort study | Prospective, observational study using telesurveillance technology to automatically track the variability in voluntary movement paths of assisted living facility residents to predict falls | This study demonstrated that the accuracy of fall prediction could be improved by combining existing risk factors with the dynamic quantitative assessment of the variability of movements. Fractal D, a measure of path tortuosity, can be used as an independent predictor of falls. |
| Rifkin et al [62] | 2013 | I | Randomized controlled trial | Randomized controlled clinical effectiveness trial; the development of a novel home-based intervention protocol using a Bluetooth-enabled blood pressure monitoring device | The inability to maintain accurate logs of blood pressures constitutes a significant barrier to evaluate response to treatment. The proposed home monitoring system improved data exchange numbers significantly in elderly people with chronic kidney disease and uncontrolled hypertension. |
| Lorenz and Opperman [63] | 2009 | V | Case report | 3 different user interfaces were developed and evaluated to monitor vital parameters | This study describes the user interface design and comparative evaluation of the interfaces. The evaluation demonstrated the acceptance of a mobile system developed for elderly people to monitor vital parameters. |
| Mihailidis et al [64] | 2008 | IV | Quasi-experiment | The evaluation of home monitoring technology acceptance | The study evaluated baby boomers’ and elderly people’s home monitoring technology preferences. The majority of the participants found these technologies acceptable. |
| Vermeulen et al [65] | 2013 | IV | Quasi-experiment | The development and evaluation of a mobile interface to monitor elderly people’s physical functions | The authors identified user requirements and asked elderly people’s preferences during the development and design of the mobile interface and evaluated the usability in a laboratory environment. The study concluded that the process resulted in an interface with good usability. |
| Merilahti et al [18] | 2009 | IV | Quasi-experiment | The evaluation of a telehealth system and comparison of working-age and community-dwelling elderly people’s usability acceptance | The study evaluated the usability, compliance, and technical feasibility of a monitoring system that comprised wearable and ambient technologies and determined 70% to 90% data collection rate. Various technical and practical problems were determined such as thunder storm damage to equipment and scheduling difference between staff and participants. |
| Palumbo et al [40] | 2014 | V | Case report | The development of a sensor network infrastructure for home care for everyday activity and long-term physiological data monitoring | GiraffPlus system that includes sensors and high-level reasoning component was implemented to support independent living for the elderly and deployed in homes across Europe for the long-term monitoring of physiological data. |
| Khan et al [66] | 2010 | V | Case report | This study proposes a low-cost sensor to continuously monitor long-term mobility as an indicator of health status in the elderly | The use of wearable accelerometers to monitor long-term activity can be used to monitor elderly people’s health status. The participants collected data without any supervision, and the proposed system provided very accurate and efficient results with little interference. |
| Demiris et al [67] | 2008 | IV | Quasi-experiment | This study evaluates focus group sessions to assess elderly people’s perceptions of specific smart home technologies | The researchers explored elderly people’s expectations of specific smart home technologies and determined a positive attitude toward sensor technologies. Most participants perceived it as useful and stated that they would agree to have these technologies in their home. |
| Demiris et al [16] | 2009 | IV | Quasi-experiment | This study explores elderly people’s privacy considerations for remote monitoring apps. | A total of 10 independent retirement community residents reviewed recorded video and expressed no privacy concerns with silhouette images. The participants expressed a desire to have control of the system operation by being able to turn it on and off. |
| Mahoney et al [68] | 2009 | IV | Quasi-experiment | The implementation and evaluation of the Automated Technology for Elder Assessment, Safety, and Environment remote home monitoring system | The families found the remote home monitoring system easy to use and satisfactory. The authors recommended a customized system that could address the users’ and stakeholders’ concern, such as limited but significant alerts. |
| Essen [69] | 2008 | IV | Quasi-experiment | The study explores senior caretaker’s electronic surveillance experience. | In-depth interviews with 17 seniors demonstrated participants’ positive experience. |
| Rantz [70] | 2013 | IV | Quasi-experiment | The study evaluates passive sensor networks deployed in independent living apartments to detect early warnings. | The authors developed alerts from sensor data recorded before emergency room visit or hospitalization, using retrospective analysis method, and evaluated the clinical relevance of alerts to enhance clinical decision making. |
| Sadasivam [43] | 2014 | IV | Quasi-experiment | Feasibility study to assess home hazard assessment for fall prevention using a remote-controlled robot | Fall risk assessment using a remotely maneuverable robot was assessed in 9 homes. Despite the fact that the researchers found little agreement between the robot and in-person video assessment and identified several technical problems, the robot video assessment determined more hazards than the in-person assessment. |
| Bourke et al [23] | 2012 | V | Case report | The eCAALYX^d^ telemedicine project was funded by the European Commission to develop and deploy fall detection, energy expenditure, and activity classification algorithms. | The researchers developed a fall detection, energy expenditure, and activity classification algorithm and successfully tested for 28 days. The study recommends further development to improve communication reliability. |
| Junnila et al [21] | 2010 | IV | Quasi-experiment | The development and evaluation of home area sensor network to monitor the elderly and homecoming after hospitalization period. | The authors used sensors to measure activity along with medical sensors and conducted tests in real homes. Capacitive floor sensors showed promise for activity monitoring, and the biggest challenge was noted as the lack of interoperability with commercial sensors. |
| Hoof and Kort [71] | 2008 | IV | Quasi-experiment | The development of Zigbee-based unattended autonomous surveillance system that includes automatic fall detection, wandering detection, and prevention | The authors developed an unattended autonomous surveillance system in the Netherlands and evaluated participants’ perceptions. The results showed a positive attitude toward the system and determined the need for additional home modifications to support aging-in-place. |
| Lai et al [72] | 2009 | IV | Quasi-experiment | The study evaluates the effectiveness of a remote training system for elderly patients | The study describes the evaluation of Remote Patient Education training architecture in the context of Informatics for Diabetes Education and Telemedicine project. The study demonstrated significant improvements in participants’ ability to effectively perform tasks and the effectiveness of remote training tool for elderly patients. |
| Courtney et al [10] | 2008 | IV | Quasi-experiment | The study investigated the adoption of smart home technology by elderly people living in independent and assisted living continuing care retirement communities. | The researchers evaluated participants’ self-perception of health, physical condition, mental and emotional condition, anticipatory living, the influence of family and friends, the influence of health care professionals, the physical environment, the technology type, and the perceived redundancy of the technology and concluded that there was a need for further exploration. |
| Wada et al [73] | 2010 | IV | Case report | This study investigates social, physiological, and psychological effects of robot therapy | The researchers developed a manual for robot therapy to provide a standard care to participants and observed participants’ responses along with caregivers’ behaviors. The study highlighted the need for further experiments. |
| Roger et al [74] | 2012 | IV | Case report | The study investigates robot-assisted therapy’s impact on dementia-related illnesses. | The researchers evaluated the benefits of social commitment robots and reported positive changes such as improvement in mood and recommended the use of similar robots for patients with dementia who experience distress and agitation. |
| Claes et al [8] | 2015 | II | Quasi-experiment | The study explores attitudes and perceptions of older adults toward contactless in-home monitoring. | This study explores the attitudes and perceptions of elderly people toward contactless monitoring using quantitative research methods using a validated questionnaire; 245 participants completed the survey and reported their willingness to share the information collected with caregivers. The authors also determined various concerns. |
| Flynn et al [24] | 2003 | V | Case report | This study explores the feasibility of virtual reality technology for patients with dementia. | The researchers assessed the physical and psychological well-being of persons with dementia while interacting with virtual reality technology and demonstrated that this technology was an appropriate medium to assess functional behavior. |
| Tapus et al [75] | 2009 | V | Case report | This study explores dementia patients’ response to a humanoid robot designed to encourage cognitive and physical activities. | The researchers designed a hypothesis-testing experiment to improve the participants’ level of attention through a cognitive stimulation game and collected results over 8 months. The participants preferred embodied socially assistive robot over the computer interface. |
| Steggell et al [76] | 2010 | IV | Quasi-experiment | The study explores the attitudes and values of immigrant older women toward assistive technologies. | The researchers developed a conceptual model of use of gerotechnology using life-span theory of control and congruence model of a person and environment interaction and conducted surveys about the application of technologies to age-in-place. They determined positive themes such as willingness to try new technologies and appreciation of the potential for improving communication with family members and negative themes such as financial concerns and language barriers for usability. |
| Matlabi et al [77] | 2012 | II | Quasi-experiment | This study explores elderly peoples' (who live in extra care housing) awareness and attitudes toward home-based technological devices. | This study analyzes elderly peoples’ (who live in extra care housing) awareness and attitudes toward basic devices including kitchen appliances and assistive technologies such as personal computers, property exit sensors, electric window openers, automatic temperature thermostats, telehealth facilities, and closed-circuit televisions. The researchers determined that participants were enthusiastic regarding the use of telecare services but expressed concerns about the potential negative impact, such as reduced social interaction. |
| Gibson et al [78] | 2015 | II | Quasi-experiment | This study explores levels of awareness and usage of assistive technology. | The researchers interviewed dementia patients about everyday use of technology and determined that their attitudes were generally positive for technologies that could facilitate independence and autonomy. |
| Niemeijer et al [79] | 2015 | II | Quasi-experiment | This study explores dementia patients’ experience with surveillance technology. | The researchers designed an explorative study in nursing homes and residential care settings and evaluated resistance to technology and patients’ patterns to cope with new space. The findings determined that surveillance technology had the potential to increase the autonomy of patients only when there was a person-centered approach. |
| Miskelly [80] | 2004 | IV | Case report | The study tests equipment designed to monitor wandering dementia patients. | The researchers developed an electronic tagging system to improve wandering dementia patients’ safety, and the equipment demonstrated excellent performance. |
| Kanoh et al [39] | 2011 | IV | Case report | This study explores the recreational robot-assisted activity for elderly people. | The researchers developed a robot-assisted activity program that comprised quizzes, tongue twisters, and arithmetic calculations for elderly people and analyzed participants interaction and reaction. The reactions were found mostly positive, and the use of this program for emotional and recreational therapy was recommended. |
| Bewernitz et al [81] | 2009 | IV | Case report | The study explores the feasibility of a machine-based prompting device for patients with moderate cognitive impairment. | The researchers an artificially intelligent system that comprised 9-step verbal prompting device and determined that participants were largely able to complete the task with machine-delivered cognitive assistance. |
| Fontecha et al [82] | 2013 | IV | Quasi-experiment | The study evaluates a centralized mobile system’s effectiveness to conduct elderly frailty assessment. | The researchers developed a framework and mobile phone app to provide frailty assessment for new incoming elderly patient. The study emphasized the need to generate an absolute frailty index that could determine the frailty condition of elderly patients. |
| Demiris et al [11] | 2013 | IV | Quasi-experiment | This study explores the effectiveness of a telehealth kiosk system. | The researchers evaluated the effectiveness of a telehealth kiosk that can assess blood pressure, weight, pulse oximetry, and blood glucose and educate patients using videos and Web-based questionnaires. The study did not find any privacy concern, but some usability issues were noted. The participants expressed an increased preference for social interactions. |
| Meiland et al [83] | 2012 | IV | Quasi-experiment | This study evaluates a day navigator’s usability in community-dwelling persons with dementia. | The evaluation determined that carers and dementia patients valued the day navigator as a user-friendly app. |
| Mihailidis et al [84] | 2008 | IV | Quasi-experiment | This study evaluates the efficacy of a prompting system to assist dementia patients through handwashing. | The AI system designed to autonomously guide elderly people with dementia showed promise on elderly people with moderate-level dementia. The researchers determined the improvement areas and emphasized the need for clinical trials. |
| Gellis et al [27] | 2014 | I | Randomized controlled trial | This randomized controlled trial evaluates a custom telehealth model’s efficacy. | The researchers designed a randomized controlled trial to compare a custom telehealth system’s effectiveness. They compared 2 groups at baseline and 3 and 6 months later. One group received the I-TEAM^e^ intervention that comprises telemonitoring, care management, and problem-solving treatment for comorbid depression. Other group received usual care plus psychoeducation. I-TEAM group had significantly fewer emergency department visits and the telehealth intervention improved their problem-solving skills. |
| Matthews et al [85] | 2015 | IV | Quasi-experiment | This study describes the evaluation of a wearable wireless camera designed to capture the challenges of dementia caregiving. | This study demonstrated the willingness of family caregivers of persons with dementia to use the novel wearable camera system to collect evidence of difficult behaviors. |
| Robinson et al [86] | 2009 | IV | Quasi-experiment | This study describes the development of a global positioning tracking system to facilitate dementia patients’ independence. | The researchers developed custom prototype tracking devices for patients with dementia and their caregivers and demonstrated the effectiveness of these technologies. Participants emphasized the preference for disguised devices. |
| Hervas et al [33] | 2014 | V | Case report | This study proposes a model focused on points of interest of well-known places for cognitively impaired people. | The researchers developed a novel mobile assistive system to support cognitively impaired people’s daily activities and demonstrated the validity in the majority of cases. |
| Marjorie et al [87] | 2009 | IV | Quasi-experiment | This study investigates sensor networks’ effectiveness to capture and recognize activity patterns to monitor elderly people. | The researchers developed a passive network system to capture physical and cognitive health conditions and determined the biggest technical challenge as the connection of sensor data to medically relevant events. |
| Astell et al [88] | 2010 | IV | Quasi-experiment | This study evaluates a custom multimedia computer system’s effectiveness to support relationships between dementia patients and their caregivers. | The researchers developed a multimedia computer system to promote dementia patient’s interaction and demonstrated that touch screen system was engaging for people with dementia. |
| We et al [89] | 2014 | IV | Quasi-experiment | This study investigates acceptance of an assistive robot using a mixed method approach. | The researchers demonstrated that participants who performed tasks to simulate robot use and those who interacted with assistive robot showed the same low intention to use the robot and did not perceive it as useful. This study identified several barriers to robot acceptance, including the feeling of stigmatization and societal issues. |
| Hewson et al [90] | 2013 | V | Case report | This study describes the development of a monitoring system for physical frailty detection. | The authors describe the development of a new system to objectively quantify the level of frailty remotely, using a Grip-ball and smartphone equipped with triaxial accelerometer received encouraging results. |
| Prescher et al [91] | 2012 | IV | Quasi-experiment | This study describes the development of eCAALYX telemedicine system to monitor elderly people with comorbidity. | eCAALYX project funded by the European Commission to prevent and manage chronic conditions of elderly people and develop ICT-based solutions. This study summarizes the development and deployment of a trial system. The authors described usability, functionality, stability, and data transmission challenges and the need for improvements. |
| Pino et al [92] | 2015 | IV | Quasi-experiment | This study evaluates elderly people’s opinion toward socially assistive robots. | The researchers designed a mixed method study to investigate socially assistive robots’ appearance, social ability, potential applications, perceived usefulness, and intentions to use and determined the importance of customization. |
| Rantz et al [93] | 2015 | IV | Quasi-experiment | This study describes the development of an unobtrusive, in-home sensor to monitor and detect elderly people’s fall risks. | The fall detection system that comprised a pulse-Doppler radar and 2 cameras was successfully tested and validated. The system is currently deployed and operating real time in several locations. |
| Mertens et al [14] | 2015 | IV | Quasi-experiment | This study focuses on the influence of attitudes toward technology on adherence. | The study evaluated 17 participants’ attitudes toward a custom telemonitoring system and determined human-machine interactions problems; 6 participants had relatively negative attitudes, and 6 had neutral attitudes toward technology. The researchers described the effect of users not needing to buy a new device before not fully exploiting all functions as amortization barriers and recommended the consideration of this barrier before prescription of telemedical systems as part of the medical therapy. |
| Torkamani et al [94] | 2014 | I | Randomized controlled trial | This study describes a multicenter randomized controlled pilot study to evaluate Aladdin telemedicine platform for assisted living. | The researchers piloted a randomized controlled pilot trial at 3 European sites to assess the quality of life of the carers and showed a significant improvement and reduction in carer burden and distress. |
| Mehrabian et al [17] | 2014 | IV | Quasi-experiment | This study evaluates cognitively impaired patients’ and their caregivers’ acceptance of home telecare technologies. | The authors described the objectives and characteristics of the telecare system using understandable concepts and conducted semistructured interviews to determine the perceptions of patients with mild cognitive impairment. The participants generally agreed that home telecare products could significantly improve the quality of life but shared some ethical and technical concerns. |
| Alexander et al [95] | 2011 | IV | Quasi-experiment | This study evaluates the effectiveness of passive sensor technology interface to assess elderly people’s activity. | The researchers evaluated content, navigation, architecture, and aesthetic appeal of infrared motion sensors and assessed usability. The study identified the need to design custom interfaces for older adults and demonstrated participants’ willingness to share their sensor data information with their families and providers. |
| Takahashi et al [96] | 2012 | I | Randomized controlled trial | This multisite randomized controlled trial evaluates the outcome of a telemonitoring study to prevent hospitalizations and emergency department visits. | The researchers implemented a home telemonitoring program to improve the cognitive status and quality of life of elderly people and evaluated the outcomes of daily telemonitoring sessions. The study concluded that telemonitoring sessions did not lower hospitalizations and emergency department visits. |
| Reder et al [97] | 2010 | IV | Quasi-experiment | This study evaluates the outcomes of a pilot remote monitoring system that uses a new sensor technology | The researchers evaluated a new sensor technology developed by Intel Labs to gather information about the daily activities of elderly people and demonstrated potential value for supporting independence. The participants were generally satisfied with the technology and reported an increase in their perceived safety, well-being, and peace of mind. The authors recommended a study with a larger sample size to validate the results. |
| Kleinberger et al [98] | 2007 | V | Case study | This study evaluates the usability and suitability of Ambient Assisted Living laboratory interfaces. | The researchers evaluated the validity of the application model of ambient intelligent care and assistance system, the adequacy of assistance settings, and accessibility of specific human-computer interfaces in the Assisted Living Laboratory and determined that elderly people would not accept information technology solutions when they do not address the real needs and are not easily accessible. |
| Frennert et al [12] | 2013 | IV | Quasi-experiment | This study evaluates elderly people’s perceptions of the GiraffPlus telehealth care system | This study evaluates elderly people’s perceptions of the GiraffPlus telehealth care system. Elderly people who were unstable when walking and with ≥1 chronic health conditions tested the system in a lab environment and reported their perceptions. The participants indicated that they would prefer face-to-face interaction with family and friends but would use the telepresence robot as well. The study demonstrated the importance of the system’s ability to support autonomy in daily life. |
| Jamsa et al [99] | 2014 | IV | Quasi-experiment | This study evaluates the effectiveness of a prototype automatic fall detection system in experimental falls. | The researchers developed a prototype waist-worn fall detection system and tested in experimental tests with young adults. The results validated the data collection of the new fall detector prototype and defined the sensitivity and specificity of different fall detection algorithms. |
| Cesta et al [38] | 2011 | IV | Quasi-experiment | This study describes the implementation of an AI robotic system. | The researchers developed an AI-based technology for domestic assistive services and used it to monitor elderly people’s activity patterns. The general attitude toward the robot was positive but multifaceted. |
| Cheng and Zhuang [25] | 2010 | V | Case study | This study evaluates the feasibility of a Bluetooth-enabled in-home patient monitoring system. | The researchers developed a Bluetooth-enabled patient monitoring system to facilitate early detection of Alzheimer disease and demonstrated the feasibility of the system. The system was able to track and record the patient location and movement patterns with success. |
| Leuty et al [100] | 2013 | IV | Quasi-experiment | This study describes the development of the Engaging Platform for Art Development to promote well-being of patients with dementia. | The researchers developed artificially intelligent touch screen art therapy devices that estimated the patient’s engagement levels and provided motivating prompts when needed and evaluated the usability. All participants found the device engaging, but they determined that the prompts were not effective. |
| Botella et al [9] | 2009 | IV | Quasi-experiment | This study describes the development of an eHealth^f^ platform designed to monitor mood, manage reports, send alert, and train and treat elderly patients. | This research was designed to present an overview of Butler eHealth platform. The authors concluded that the use of the system increased positive emotions and decreased negative ones, and satisfaction level was high. |
| Lin et al [101] | 2008 | IV | Quasi-experiment | This study describes the development of a dementia patient safety maintenance information platform that comprises a dementia assessment and safety maintenance modules. | The researchers developed an information platform to improve dementia patients’ safety during outdoor activities, implemented at a daytime care organization and determined response times and patient compliance. |
| Chen et al [26] | 2011 | V | Case study | This study introduces a new Web-based home monitoring platform for Parkinson disease patients. | In this study, researchers share their findings during the test phase of a new wearable sensor-based monitoring system for Parkinson disease patients. |
| Pollack et al [41] | 2002 | V | Case study | This study describes the development of a mobile robotic assistant to remind elderly people routine activities and help navigate their environments. | The researchers share their experience with the development of a Nursebot app. |
| Demiris et al [13] | 2008 | IV | Quasi-experiment | This study evaluates the findings of a smart home project in a retirement facility. | The researchers evaluated the adoption and acceptance of sensor technologies and determined that the residents did not change their routines because of the sensors. |
| Surie et al [102] | 2008 | V | Case study | This study describes the experimental setup of a Zigbee communication protocol–based wireless sensor network. | The researchers developed a wireless smart home sensor network to keep track of users’ interaction with objects and determine their situations. They determined an overall precision value of 91.2% and concluded this outcome as promising. |
| Hori et al [28] | 2004 | V | Case study | This study describes the development of an ultrasonic network system to monitor elderly people in nursing homes. | The researchers installed the Ultra Badge System to monitor elderly people’s position and determined that the system had the potential to detect accident-prone events in advance. |
| Do et al [19] | 2018 | IV | Quasi-experiment | This study describes the development of a robot-integrated smart home testbed. | This study describes the experimental infrastructure and software architecture of a RiSH^g^. The participants conducted daily activities in the testbed and mimicked falls, and RiSH was able to detect falling sounds with an accuracy of 80%. The authors recommend further tests. |
