# Supplementary material for: Innovative Assisted Living Tools, Remote Monitoring Technologies, Artificial Intelligence-Driven Solutions, and Robotic Systems for Aging Societies: Systematic Review
Source: JMIR Aging. 2019 Nov 29;2(2):e15429. doi: 10.2196/15429 (PMC6911231; doi:10.2196/15429)
Supplement: Multimedia Appendix 2 [file aging_v2i2e15429_app2.docx]

| **Main groups of technology solutions** | **Author [reference]** | **Novel technology and innovation** |
| --- | --- | --- |
| **Technology acceptance and readiness** | Botella et al [9] | Butler eHealth^a^ platform that comprises 3 platforms: (1) user platform is designed for the elderly person, (2) professional user platform is designed for the health agents, and (3) external user platform is designed for family and friends; each platform includes diagnostic, therapeutic and playful apps. |
|  | Wild et al [45] | N/A^b^ |
|  | Claes et al [8] | Contactless motion, temperature, pressure, and bed sensors; video cameras; public utility sensors (water, gas, and electricity) |
|  | Courtney et al [10] | Bed, motion, kitchen safety, and fall detection sensors |
|  | Demiris et al [11] | Multi-user telehealth kiosk |
|  | Demiris et al [13] | In-home monitoring system: wireless infrared proximity sensors to detect motion; pressure switch pads; stove temperature sensors; sensors on cabinet doors; bed sensors; respiration, pulse, and movement sensors; data manager to collect data from the sensors |
|  | Demiris et al [16] | Vision-based recognition methods that anonymize images by extracting only a silhouette |
|  | Frennert et al [12] | GiraffPlus telehealth care system: blood pressure, bed/chair occupancy sensors; semiautonomous telepresence robot |
|  | Mehrabian et al [17] | N/A |
|  | Merilahti et al [18] | IST WristCare wireless activity monitor; electromechanical film sensor to collect vital signs during bed time; wellness diary mobile phone app; temperature and illuminations sensors; Omron pedometer; blood pressure monitor; body weight scale |
|  | Mertens et al [14] | Panasonic Toughbook (CF-H1); wireless weight scale; Beurer BS 9930 BT; wireless blood pressure monitor; Boso Medicus Prestige; wireless electrocardiogram BioHarness chest band |
|  | Mihailidis et al [15] | Personal emergency response system; automated fall detection; lifestyle monitoring; activity of daily living prompting; environmental controls; health and physiological monitoring; interactive video teleconferencing |
| **Novel patient monitoring and smart home technologies** | | |
|  | Bourke et al [23] | The mobile Enhanced Complete Ambient Assisted Living Experiment monitoring system that consists of a wearable body sensor system that includes skin temperature, respiratory rate, mobility heart rate, fall and activity monitoring sensors; caretaker server and electronic health record server |
|  | Do et al [19] | Home service robot built on a Pioneer P3-DX base; touch screen monitor for videoconferencing; laser rangefinder; Asus Xtion Pro Live RGB^c^ and Depth camera (vision system); microphone array of the PS3eye camera (auditory system); wearable unit comprising physiological sensors, motion sensors, a smart watch, and wearable eHealth Sensor Platform v2.0 |
|  | Elakkiya et al [20] | Data collected from CASAS smart home test bed; context-based clinical score assessment that measures the following scores: duration, number of events occurred, activity recognition, activity sequence, activity interruption, and activity parallelism |
|  | Junnila et al [21] | Zigbee network technology; weight scale, video call, blood pressure monitor, infrared, and bed and floor sensors |
|  | Cheng and Zuang [25] | Bluetooth access points in every room of the house; Hewlett-Packard iPAQ pocket PC; Microsoft structured query language server |
|  | Chen et al [26] | MercuryLive home monitoring platform—software services: central server and patient’s and clinician’s hosts; Web-based graphical user interface (GUI) client with videoconferencing; body sensor network |
|  | Ferreira et al [22] | eXtreme Programming agile software development method |
|  | Fontecha et al [82] | Mobile phone app; accelerometer; server |
|  | Flynn et al [24] | Onyx 1 computer that can run real-time models with a high degree of details; Belgian American Radio Corporation 128 projectors which provide semi-immersive view |
|  | Gellis et al [27] | In-home monitor for weight, blood pressure, oxygen saturation, and temperature |
|  | Hewson et al [90] | Grip-ball wireless pressure measurement system; smartphone with triaxial accelerometer; wireless digital bathroom scale |
|  | Hori et al [28] | Ultra Badge system: 100 ultrasonic receivers on the ceiling; signal generators; radio frequency transmitter; ultrasonic 3D^d^ tags; host computer |
| **Intelligent algorithm development and software engineering** | | |
|  | Kurnianingsih et al [30] | Body temperature and heart rate sensors; wearable display; context-aware framework that comprises the following components: human context, environment context, learning context, and wearable context |
|  | Cook [29] | MavHome software architecture to monitor and provide automated assistance; motion, light, temperature, humidity, smoke, gas sensors; PostgreSQL database for data mining and prediction; Multiagent technologies |
|  | Ge et al [31] | Pyroelectric sensor |
|  | Hervas et al [33] | Software app that provides spatial guidance; daily tasks reminder; monitoring app |
|  | Wang et al [34] | Ontology-based formal context model: extensible context ontology |
|  | Jeon et al [35] | RGB camera; 3D depth camera; microphones installed in smart home or care facility; care knowledge sharing system programmed in Java |
|  | Zhang et al [32] | Prediction models for mobile phone–based video streaming system |
| **Robotics technologies** | | |
|  | Cesta et al [38] | RoboCare domestic robot |
|  | Filan and Llewellyn-Jones [104] | Artificial intelligence bot dog robot |
|  | Roger et al [74] | Paro, socially interactive seal robot |
|  | Wada et al [73] | Paro, the seal robot with tactile, light, and posture sensors; speaker; and microphones |
|  | Libin and Cohen Mansfield [42] | Emotional communication robot NeCoRo (robotic cat) |
|  | Chivarov et al [36] | Mobile robot; intelligent control systems; tactile, infrared, and ultrasound sensors; voice recognition and generation systems |
|  | Kanoh et al [39] | Yorisoi Ifbot |
|  | Palumbo et al [40] | Giraff telepresence robot; GirffPlus sensor network: blood pressure monitor, glucose, temperature, weight, and oxygen sensors; information management and storage management software |
|  | Pollack et al [41] | Pearl autonomous Nursebot: Autominder; speech recognition and synthesis software; fast image capture and compression software for video streaming on the Web; face detection and tracking software |
|  | Sadasivam et al [43] | Spykee robot |
|  | Ramoly et al [37] | Nao H25 humanoid robot; open source development framework to manage smart homes; motion sensors, opening sensors, thermometers, and beacons; Java, C++, Python |
